# Wheat Domestication Accelerated Evolution and Triggered Positive Selection in the β-Xylosidase Enzyme of *Mycosphaerella graminicola*

**DOI:** 10.1371/journal.pone.0007884

**Published:** 2009-11-18

**Authors:** Patrick C. Brunner, Nicolas Keller, Bruce A. McDonald

**Affiliations:** Institute of Integrative Biology, Plant Pathology Group, ETH Zurich, Zurich, Switzerland; University of Texas Arlington, United States of America

## Abstract

Plant cell wall degrading enzymes (PCWDEs) of plant pathogens are receiving increasing interest for their potential to trigger plant defense reactions. In an antagonistic co-evolutionary arms race between host and pathogen, PCWDEs could be under strong selection. Here, we tested the hypothesis that PCWDEs in the fungal wheat pathogen *Mycosphaerella graminicola* have been positively selected by analyzing ratios of non-synonymous and synonymous nucleotide changes in the genes encoding these enzymes. Analyses of five PCWDEs demonstrated that one (β-xylosidase) has been under strong positive selection and experienced an accelerated rate of evolution. In contrast, PCWDEs in the closest relatives of *M. graminicola* collected from wild grasses did not show evidence for selection or deviation from a molecular clock. Since the genealogical divergence of *M. graminicola* from these latter species coincided with the onset of agriculture, we hypothesize that the recent domestication of the host plant and/or agricultural practices triggered positive selection in β-xylosidase and that this enzyme played a key role in the emergence of a host-specialized pathogen.

## Introduction

The antagonistic co-evolution caused by host-pathogen interactions is among the most important forces shaping organismal diversity. In plants, natural selection has generated a complex array of defense mechanisms to prevent or limit infection by pathogens. The basal immune system of plants detects molecules that are broadly conserved across a wide range of pathogen taxa. These molecules have been named pathogen-associated molecular patterns (PAMPs). Detection of microorganisms induces PAMP-triggered immunity (PTI), which may be the plant's first line of defense. By definition, PAMPs form part of vital structures or functions of pathogens, hence, it is generally believed that they cannot be lost or significantly modified without reducing pathogen fitness [1]. Previously identified PAMPs that elicit PTI in plants include fungal chitin and bacterial flagellins [2], [3]. A second line of more specific resistance is induced in “gene-for-gene” interactions referred to as effector-triggered immunity (ETI). ETI involves proteins encoded by the plant resistance (R) genes that detect pathogen effector proteins.

Co-evolution in a pathogen-plant interaction is characterized by the development of resistance in the host and virulence in the pathogen, with natural selection affecting the type of genetic polymorphism found at a selected locus. Positive diversifying selection is an evolutionary force that favors new mutations conferring a fitness advantage and thus leads to an increase in gene diversity and accelerates divergence between homologous proteins. Positive selection has been detected in R-genes of plants and effector genes of plant pathogens. Among the pathogen effector genes shown to be under positive selection are genes involved in the type III secretion system of bacteria [4], fungal avirulence genes [5], and genes encoding host-specific toxins [6], [7]. Plant cell wall degrading enzymes (PCWDEs) of plant pathogens are receiving increasing interest for several reasons [8]. PCWDEs are essential for plant pathogens to digest plant cell walls and release nutrients from the cell wall polysaccharides [9]. PCWDEs have also been identified as significant fungal pathogenicity components [10], [11], [12]. Because PCWDEs or some of their degradation products may elicit defense responses in plants [13], they could also function as elicitors of plant innate immunity.

Plant produced inhibitors of PCWDEs such as polygalacturonase-inhibiting proteins (PGIPs) have been known for decades and were proposed to form part of the plant immune system (reviewed in [14]). In recent years a growing number of plant proteins have been isolated that inhibit the activity of PCWDEs such as endoxylanases, including TAXI *Triticum aestivum* xylanase inhibitor [15] and XIP xylanase inhibitor protein [16]. This growing evidence for a complex array of PCWDE inhibitors provides compelling evidence for another avenue of a plant–microbe co-evolutionary arms race [17] that is similar to the ETI gene-for-gene systems, but in this case the inhibitor proteins of the plant interact with hydrolytic enzymes produced by the pathogen.

*Mycosphaerella graminicola* (anamorph *Septoria tritici*) is a widespread fungal pathogen causing septoria tritici leaf blotch disease of wheat. *M. graminicola* is host specific [18], [19] affecting mainly cultivated wheat (*Triticum aestivum*). Recently, two studies provided the first reports of *in vitro* PCWDE production for *M. graminicola*. Some of these enzymes (β-xylosidase, polygalacturonase and xylanase) showed a significant correlation between *in vitro* production and disease symptoms, including leaf area covered by necrotic lesions and pycnidia, suggesting that these PCWDEs could be important determinants of pathogenicity in *M. graminicola* [20], [21]. Fungal xylanases from *Trichoderma* spp. were also identified as potent elicitors of defense responses in various plants [22]. The general importance of endo-xylanases produced by microbial plant pathogens was reviewed by Beliën et al. [8]. Given the earlier findings, we hypothesized that some pathogen PCWDEs (such as xylanase) have been involved in an antagonistic coevolution with their plant host through their activity as PAMPs or effectors or through interactions with plant inhibitors. The general objective of this study was to quantify selection on PCWDEs using the *M. graminicola*-wheat model.

Natural selection at the molecular level can be statistically distinguished from patterns of neutral evolution and genetic drift by using likelihood-based methods and inferring ratios of non-synonymous to synonymous mutations. Directional, purifying selection occurs when successive amino acid changes (i.e. non-synonymous changes) improve protein efficacy and consequently the changes tend to be fixed in future lineages, thereby reducing overall genetic diversity. For example, the amino acid sequence that is optimum for β-xylosidase activity in wheat may not be optimum in barley, hence purifying selection on the different hosts may lead to fixed allele differences in pathogen populations that become specialized on different hosts. On the other hand, positive selection occurs when several phenotypes are favored, for example under the scenario of antagonistic coevolution whereby effectors evolve to avoid detection by host receptors or enzymes evolve to avoid inactivation by host-produced inhibitors, resulting in an excess of non-synonymous mutations and an overall increase in genetic diversity.

Phylogeographic studies and migration analyses based on DNA data suggested that the center of origin of *M. graminicola* was the Middle East and that the pathogen was dispersed throughout the world during the spread of wheat cultivation [23], [24], [25]. Stukenbrock et al. [26] tested the hypothesis that *M. graminicola* emerged as a pathogen during the domestication of its wheat host by comparing gene genealogies of *M. graminicola* with closely related populations collected from uncultivated grasses in Iran, the center of origin of both host and pathogen. Their results provided strong evidence that *M. graminicola* originated in the Middle East approximately 10,500 years ago, mediated by the wheat domestication process, from sympatric ancestral populations infecting wild grasses. During this process, *M. graminicola* emerged from a species complex of generalist pathogens that infect many grass genera to become a specialist pathogen infecting wheat. This plant pathogen system provides a unique opportunity to address two questions using phylogenetically based analyses, including: 1. Do PCWDEs show molecular evidence of positive selection supporting the hypothesis of a co-evolutionary arms-race between plant and pathogen? 2. Were the enzymes involved in cell wall degradation also affected by this host specialization, leading to purifying selection and/or varying rates of nucleotide substitutions in their coding genes?

## Results

Sources of *Mycosphaerella graminicola* isolates included in this study are summarized in Table S1. In total, 189 field-collected strains of *M. graminicola* originating from Iran, Israel, Oregon, Switzerland and Germany were sequenced for five genes encoding PCWDEs. Sequence data from the non-coding RFLP locus STS2 used in previous studies [24], [25] were added to the data set for comparison. To address the question of genetic divergence among host adapted populations, we also included isolates of *Mycosphaerella* spp. collected from uncultivated grasses of three different genera, *Agropyron repens*, *Dactylis glomerata* and *Lolium multiflorum*.

Summary statistics of genetic diversity are given in Table 1. No length variation was found in coding regions for any of the genes. Genetic diversity was compared for the wild grass-adapted lineages “S1” and “S2” and wheat-adapted *M. graminicola* isolates collected from the same locations in Iran. Both haplotype and nucleotide diversity values were significantly higher in *M. graminicola* collected from wheat for all five PCWDEs, indicating a generally faster molecular evolution for the “domesticated” wheat pathogen. The highest ratio of non-synonymous to synonymous substitutions was observed at the β-xylosidase gene in *M. graminicola* from wheat. However, the ratio varied considerably among PCWDEs, both within and among the evolutionary groups, suggesting different amounts and/or types of selection acting on the various genes.

**Table 1:** Summary statistics of exon sequence diversity for *Mycosphaerella* populations collected from wheat and wild grasses.

Enzyme	Host	*n* a	Codons^b^	S^c^	*K* ^d^	Sd^e^	π^f^
β-xylosidase	total on wheat	95	294	41	33 (12)	0.889 (0.888)	0.009
	Iran on wheat	36	294	22	8 (6)	0.679 (0.678)	0.007
	Iran on grass S1	22	294	0	1	0	0
	Iran on grass S2	22	294	0	1	0	0
Cellulase	total on wheat	108	170	49	59 (15)	0.945 (0.944)	0.021
	Iran on wheat	45	170	19	7 (5)	0.709 (0.709)	0.010
	Iran on grass S1	21	170	34	2 (2)	0.467 (0.467)	0.031
	Iran on grass S2	20	170	1	2 (2)	0.337 (0.337)	0.001
Cutinase	total on wheat	109	206	43	55 (14)	0.960 (0.959)	0.011
	Iran on wheat	46	206	30	26 (14)	0.957 (0.957)	0.012
	Iran on grass S1	22	206	8	2 (2)	0.485 (0.485)	0.006
	Iran on grass S2	18	206	7	2 (2)	0.425 (0.425)	0.005
Polygalacturonase	total on wheat	108	295	53	40 (13)	0.952 (0.953)	0.009
	Iran on wheat	48	295	14	17 (11)	0.936 (0.936)	0.006
	Iran on grass S1	22	295	27	2 (2)	0.519 (0.519)	0.016
	Iran on grass S2	17	295	1	2 (2)	0.529 (0.529)	0.001
Xylanase	total on wheat	101	220	29	49 (17)	0.968 (0.961)	0.011
	Iran on wheat	46	220	21	25 (13)	0.980 (0.975)	0.011
	Iran on grass S1	22	220	22	4 (3)	0.766 (0.765)	0.017
	Iran on grass S2	22	220	13	3 (3)	0.524 (0.524)	0.010

NOTE. Numbers in brackets are simulated values adjusted to the smallest sample size. One of the cutinase haplotypes (found in two isolates from Israel) contained a TGA stop-codon, and was hence excluded from the selection analyses.

a number of isolates, ^b^ number of codons, ^c^ number of polymorphic sites, ^d^ number of haplotypes, ^e^ haplotype (gene) diversity, ^f^ nucleotide diversity.

The hypothesis of neutral evolution was tested using three approaches. Results from Tajima's D [27] test and Fay and Wu's H [28] test failed to reject the hypothesis of neutral evolution for all five enzymes (P>0.1 for all comparisons). Both are within-species tests that compare summary statistics of allele frequency distribution against values expected under neutral evolution. Drawbacks of these tests are that they are sensitive to demographic events such as changes in population size and they may not be informative when using samples from geographically distinct populations. The second approach compared ratios of synonymous and non-synonymous substitutions within and between species using the McDonald-Kreitman tests [29]. This analysis also failed to reject the neutral hypothesis for three enzymes (Table S4). However, the β-xylosidase and cellulase data sets deviated significantly from neutral expectations. The excess of non-synonymous substitutions between *M. graminicola* and the outgroup “S1” is evidence for positive diversifying selection at β-xylosidase, while cellulase was under purifying selection. This implies that after the divergence of *M. graminicola* and the “S1” lineage from wild grasses, fixation of non-synonymous mutations occurred preferentially in the wheat-adapted *M. graminicola*.

Finally, a likelihood-ratio method implemented in the program SLR [30] was used to compare different evolutionary models against the data sets. For “S1” and “S2” no codon site under significant positive selection was detected. In contrast and supporting the results from the McDonald-Kreitman test, positive selection was inferred for the β-xylosidase data set from wheat-adapted *M*. *graminicola*, whereas likelihood ratio tests for all other genes were non-significant (Table 2; Table S3). The analyses identified five specific codon sites under significant positive selection. The same analysis was conducted separately for the Iranian isolates to compare results between sympatric populations collected from wheat and wild grasses. This analysis identified three of the five codon sites detected in the total *M. graminicola* data set as being under positive selection. Codon sites showing purifying (negative) selection were detected in all enzymes, except in β-xylosidase (Table 2).

**Table 2:** Summary statistics for likelihood-ratio-test (LRT) for detecting non-neutral sites in *Mycosphaerella graminicola* samples from wheat.

	Positive selection a		Negative selection b
Enzyme	Codon site	LRT_Stat	Pval		proportion of sites
β-xylosidase	48	5.173	0.022		0/294
	84	10.335	0.001		
	192*	13.117	<0.001		
	222*	12.659	<0.001		
	246*	6.859	0.008		
Cellulase	None	-	-		147/170
Cutinase	None	-	-		15/206
Polygalacturonase	None	-	-		5/295
Xylanase	None	-	-		64/220

NOTE. No positively selected codon sites were detected for the wild grass samples “S1” and “S2”. Asterisks denote significant positive sites for the Iran samples on wheat.

a With the exception of site 48, the same positively selected sites were detected using the program PAML (Table S3).

b Because of the large number of negatively selected sites in some enzymes, the number of selected codons per total number of codons is given.

**Figure 1:**
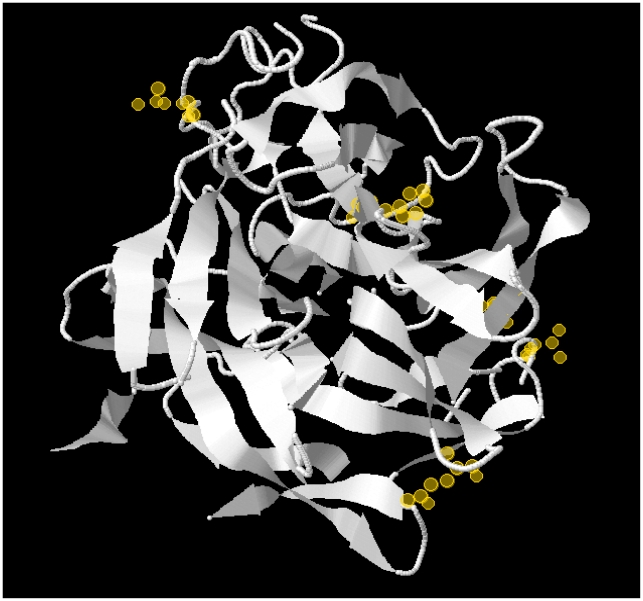
Predicted protein structure of β-xylosidase of *M. graminicola*. Note that the amino acid sites under positive diversifying selection (yellow halos) are located on the surface of the protein.

The predicted protein structure of β-xylosidase of *M. graminicola* was modeled based on multiple-threading alignments and simulations using I-TASSER [31]. The highest similarity (C-score  = −0.01) was obtained with the crystal structure of β-xylosidase from *Bacillus halodurans* C-125 (GenBank accession; 1YRZ_A). Intriguingly, all sites under positive diversifying selection are located on the surface of the protein, a prerequisite for interaction with the putative host detection-system (Figure 1).

**Figure 2:**
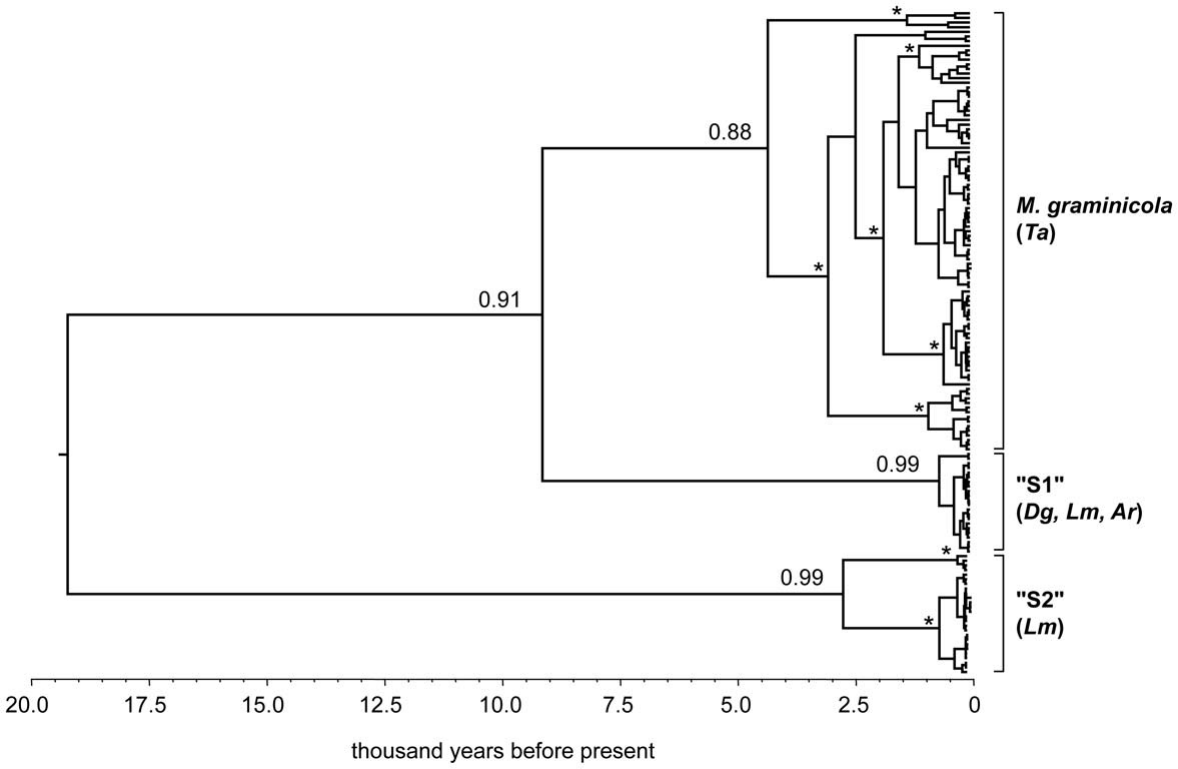
Phylogenetic relationships among fungal samples from different hosts. Maximum clade credibility (MCC) tree from Bayesian coalescent analysis depicting the phylogenetic relationships among fungal samples collected from domesticated bread wheat (Ta, Triticum aestivum) and wild grasses (Ar, *Agropyron repens*; Dg, *Dactylis glomerata*; Lm, *Lolium multiflorum*). Clade-credibility values are shown only for the three major lineages. Asterisks denote nodes within lineages that received >90% posterior support.

Phylogenetic relationships among isolates were determined using a maximum clade credibility (MCC) tree with BEAST, which implements a Bayesian coalescent approach with Markov-Chain Monte Carlo sampling [32]. The analysis distinguished three major evolutionary lineages of β-xylosidase with posterior values ≥88% (Figure 2). The same general topology was obtained for the other four PCWDEs and also for the non-coding STS2 data set (Figure S1). The largest cluster contained all *M. graminicola* samples, consistent with a complete lineage sorting between fungal isolates collected from wheat and wild grasses. The two other clusters were composed exclusively of fungal isolates collected from wild grasses. The sister-group to *M. graminicola* (“S1”) contained isolates collected from *Agropyron repens, Dactylis glomerata* and *Lolium multiflorum*, while the last group (“S2”) contained only isolates collected from Lolium multiflorum. This phylogeny corroborates previous results presented by Stukenbrock et al. [26] using different genes.

**Figure 3:**
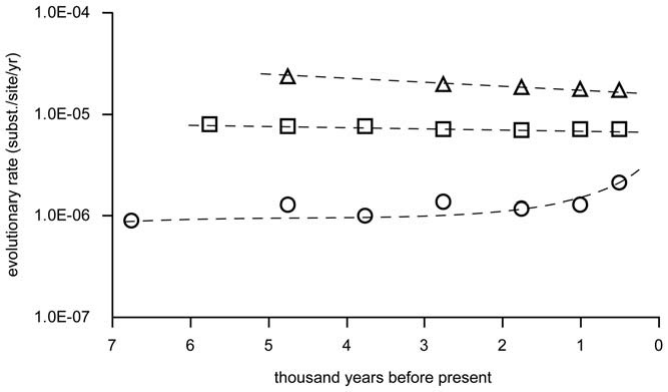
Accelerated rate of evolution in β-xylosidase. Estimates of evolutionary rates for the positively selected β-xylosidase (circles), cellulase (squares), and the non-coding “STS2” nucleotide sequence (triangles) in *Mycosphaerella graminicola.*

Deviation from a constant rate of molecular evolution within the PCWDE data sets (ie a “molecular clock”) was first assessed using the likelihood ratio test (LRT) implemented in the program HYPHY [33]. Since the number of non-synonymous substitutions in a data set may be influenced by purifying and positive selection, the LRT was applied separately on the three codon models synonymous, non-synonymous and full (both synonymous and non-synonymous) rates. Only the β-xylosidase data set deviated significantly from a molecular clock, with a consistent outcome for all three analyses (P<0.001) (Table S5). Second, Bayesian estimates of evolutionary rates along the genealogy of *M. graminicola* were calculated using the “relaxed clock method” [34] from values obtained with the genetic coalescent analysis described above. As expected, average evolutionary rates plotted as a function of time were higher for the non-coding STS2 sequence data set compared to the PCWDEs (Figure 3). Linear regression analysis revealed a statistically significant positive correlation for β-xylosidase with evolutionary time (P = 0.023). The best fit of the data, however, was obtained using a logarithmic equation (y = 0.0002Ln(x) +0.0014; r = 0.82), indicating an accelerated rate of evolution from past to present. In contrast, for all other PCWDEs and STS2 evolutionary rates remained constant or declined slightly throughout the genealogy of *M. graminicola*. This latter result is typical for most proteins as a consequence of purifying selection.

**Figure 4:**
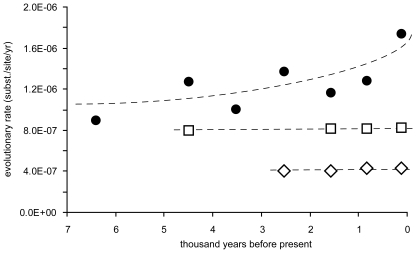
Accelerated rate of evolution in *M. graminicola.* Estimates of evolutionary rates for *Mycosphaerella graminicola* and its two closest relatives “S1 and “S2” (see Figure 2) under a relaxed clock model. Mean rates for internal branches of the β-xylosidase-based phylogeny are shown as filled circles for *M. graminicola*, squares for the lineage “S2”, and diamonds for the lineage “S1”, respectively.

β-xylosidase was the only PCWDE consistently showing positive selection using different molecular approaches. Therefore, we conducted a separate “relaxed clock” analysis for this enzyme (based on the phylogeny depicted in Figure 2) by including the sister groups “S1” and “S2”. The β-xylosidase gene of wheat-adapted *M. graminicola* showed significantly higher rates of evolution than the grass-adapted “S1” and “S2” lineages. Evolutionary rates inferred for “S1” and “S2” remained constant over time and did not show the acceleration observed in *M. graminicola* (Figure 4).

## Discussion

Analyses of genes encoding PCWDEs in *M. graminicola* and closely related species provided novel insights into the evolutionary processes associated with pathogen domestication. A comparison of synonymous and non-synonymous substitution rates demonstrated that β-xylosidase has been under strong positive diversifying selection and experienced an accelerated rate of evolution during the 10,000 years since the emergence of the “domesticated” pathogen *M. graminicola*. In contrast, PCWDEs in “wild” *Mycosphaerella* spp. collected from wild grasses did not show evidence for selection or deviation from a molecular clock. Since the genealogical divergence of *M. graminicola* from S1 coincided with the onset of agriculture [26], we hypothesize that the domestication of the host plant and/or agricultural practices associated with the cultivation of wheat triggered positive diversifying selection and accelerated evolution in β-xylosidase, suggesting that this PCWDE played a key role in the emergence of the new pathogen species that was specialized on wheat.

An alternative explanation for the observed acceleration in the rate of evolution is that the β-xylosidase gene came under relaxed selection. However, our results provide no support for this hypothesis. Population genetic theory predicts that evolutionary rates of genes can increase due to reductions in population size [35]. But Stukenbrock et al. [26] showed that the population size of *M. graminicola* strongly increased since its divergence from S1, probably due to the spread of wheat cultivation. Population size reduction is likely to affect all genes, but our results indicated accelerated evolution in only one of the five tested genes. Finally, β-xylosidase showed several codon sites under strong positive selection, which is not expected under relaxed selection. For these reasons we believe that adaptive evolution is the major mechanism explaining the increased rate of evolution in β-xylosidase.

Several evolutionary scenarios can explain our findings. First, the domestication of wheat from wild grasses was a process that created a new host species with possibly new cell wall properties. Hence, positive selection of the β-xylosidase gene could reflect functional optimization resulting in the expression of a biochemically better adapted protein, for example by altering its hydrolytic activity. Cooper et al. [36] found differences in the kind and quantity of PCWDEs produced depending on the host plant and hypothesized a host-specific adaptation through co-evolution in cereal pathogens such as *Rhizoctonia cerealis*, *Fusarium culmorum* and *Pseudocercosporella herpotrichoides*. In the absence of a three-dimensional structural model, it is not possible to associate the positively selected sites in β-xylosidase with functional domains of the protein. But if the observed positive selection was due to selection for an optimized hydrolytic activity in the new host, we would expect to find relatively few β-xylosidase haplotypes among the domesticated *M. graminicola* strains as a result of purifying selection during the 10,000 years since wheat domestication. Instead, we found eight β-xylosidase haplotypes in Iranian *M. graminicola* (33 haplotypes worldwide) compared to one haplotype in the sympatric wild S1 species that infects three grass genera. Thus we reject the hypothesis of selection for optimized hydrolytic activity on wheat.

Second, a growing number of plant proteins have been isolated that inhibit the activity of pathogen produced PCWDEs and these were proposed to form part of the plant immune system [14]. In contrast to the adaptation described above, this evolutionary process would involve an arms race between plant and pathogen [17], [37]. Plant produced inhibitors of PCWDEs such as polygalacturonase-inhibiting proteins (PGIPs) and xylanase inhibitor proteins (XIPs) have been known for decades. The majority of studies to understand the complex mechanism involved in this coevolutionary process focused on the host (e.g. [38], [39], [40]), so little is known about the pathogen PCWDEs which were the focus of our analysis. These previous studies identified xylanase and polygalacturonase as the most promising candidates for PCWDEs evolving in an “arms-race” model of co-evolution with plant defense proteins. However, we did not find evidence for positive selection acting on these genes in *M. graminicola*. Instead, our results are consistent with strong positive selection operating on β-xylosidase. The detection of plant produced inhibitor proteins acting against β-xylosidase would corroborate our hypothesis of a plant–microbe co-evolution involving this PCWDE. To our knowledge no such proteins have been described to date, but our findings provide a rationale to begin searching for plant-produced inhibitors of this enzyme.

Finally, our findings can be explained by an arms-race featuring evasion of recognition by plant immune receptors. In this scenario, β-xylosidase would be recognized by a plant receptor as an effector that activates plant defenses. The in vitro production of xylanase and polygalacturonase has been significantly correlated with necrosis in the host, suggesting that these PCWDEs are determinants of pathogenicity in *M. graminicola* [21]. Similar results showing a correlation between PCWDE production and pathogenicity have been confirmed for other fungal pathogens infecting cereals, including *Phaeosphaeria nodorum* [41], [42], *Rhizoctonia cerealis*, *Fusarium culmorum* and *Pseudocercosporella herpotrichoides* [36], [43]. *B. cinerea* is a necrotrophic fungus that possesses six polygalacturonase loci that are potentially involved in host specialization [44]. It was demonstrated that the degradation of the plant cell wall by these enzymes induced plant-signaling responses [45], [46]. In a recent study, Rowe and Kliebenstein [47] assessed the genetic variation at three polygalacturonase loci in *B. cinerea* to address the hypothesis of a pathogen-plant co-evolution. The distribution of genetic variation showed only limited evidence for host-specific subdivision. However, the loci differed significantly, with two loci exhibiting elevated genetic variation and showing evidence of positive selection. In the case of β-xylosidase, the enzyme is conserved across a broad array of fungal and bacterial pathogens, suggesting a vital function that could make it a PAMP targeted by receptors that activate PTI. β-xylosidase could also act as an effector protein that activates ETI. Surveys of natural pathogen populations have shown positive selection operating on genes encoding other effector molecules, including Avr genes and host specific toxins [4], [7], but we are not aware of any studies showing positive selection operating on PAMPs.

We propose that the selection processes operating on β-xylosidase are quite different in pathogen populations infecting wild grasses compared to wheat. The “wild” S1 *Mycosphaerella* species infects at least three different genera of grasses that exist with a patchy distribution in a heterogeneous natural habitat, while the “domesticated” *M. graminicola* species infects a single grass species in a specialized agricultural ecosystem characterized by environmental homogeneity, high host density and reduced genetic diversity. We proposed earlier that the unique characteristics of the agricultural ecosystem provide a special environment that favors the rapid emergence of host-specialized pathogen species [48]. We hypothesize that β-xylosidase is interacting either with a host receptor (ie acting as a PAMP or effector) or with an inhibitor (ie acting as a target of plant defense proteins) produced by the wheat host. In either case, it appears that this interaction arose very recently, ie in the 10,000 years since wheat was domesticated. Further analyses will be needed to fully understand the role of β-xylosidase in pathogen-plant co-evolution, but these findings show that β-xylosidase may have played a major role in the emergence of the wheat pathogen *M. graminicola*.

## Materials and Methods

### PCWDEs and Sequence Amplification

Recent *M. graminicola* studies described the production of enzymes degrading various components of plant cell walls such as xylan, cellulose or pectic polysaccharides [21]. From these tested PCWDEs we selected β-xylosidase, cellulase, polygalacturonase and xylanase, all of which expressed significant in vitro activity. We also included cutinase as a potentially important enzyme for penetrating the plant surface during the first steps of infection. Many of these enzymes belong to protein families having similar functions and structures, thus particular care was taken in designing specific primers to avoid cross-amplification of related genes. PCR primers were designed using nucleotide sequences of target genes identified in the *M. graminicola* genome project (Table S2). PCR products were sequenced and verified with the *M. graminicola* genome as well as with the nucleotide database available from GenBank (http://www.ncbi.nlm.nih.gov) using BLAST searches. Sequences of final primer pairs yielding unambiguous products are provided in Table S2.

### SLR

Evidence for non-neutral evolution was assessed by comparing the rate of non-synonymous substitutions with the rate of synonymous substitutions (dN/dS  = ω) using a likelihood-ratio-test based analysis as implemented in the SLR software package (“sitewise likelihood-ratio”; [30]). The SLR method assumes that substitutions at a given codon site occur independently of every other site and that the process can be modeled as a continuous-time Markov process. The test consists of performing a likelihood-ratio test on a site-wise basis, testing the null model (neutrality, ω = 1) against an alternative model ω≠1 (ie purifying selection ω<1; positive selection ω>1).

Additional information on “Materials and Methods” can be found in Supporting Information S1.

## Supporting Information

Supporting Information S1 Wheat Domestication Accelerated Evolution and Triggered Positive Selection in the β-Xylosidase Enzyme of *Mycosphaerella graminicola*. Additional information on samples and analyses.
(0.04 MB DOC)

Table S1 Origin of fungal isolates.
(0.04 MB DOC)

Table S2 Reference sequences of all five plant cell wall degrading enzymes identified from the *Mycosphaerella* genome project at http://genome.jgi-psf.org.
(0.05 MB DOC)

Table S3 Summary statistics for likelihood-ratio-test (LRT) using PAML for positive selection in the *Mycosphaerella graminicola* samples from wheat.
(0.05 MB DOC)

Table S4 McDonald-Kreitman test of neutrality for *Mycosphaerella graminicola* nucleotide data sets.
(0.05 MB DOC)

Table S5 Likelihood ratio testing for the assumption of a molecular clock.
(0.03 MB DOC)

Figure S1 Phylogenetic relationships among fungal samples for different enzymes. Neighbor-Joining trees (NJ) based on the PAM matrix (Dayhoff model) of amino acid sequences for the cell-wall-degrading enzymes cellulase, cutinase, polygalacturonase and xylanase.
(1.07 MB TIF)
