# Supplementary material for: Wheat Domestication Accelerated Evolution and Triggered Positive Selection in the β-Xylosidase Enzyme of Mycosphaerella graminicola
Source: PLoS One. 2009 Nov 18;4(11):e7884. doi: 10.1371/journal.pone.0007884 (PMC2774967; doi:10.1371/journal.pone.0007884)
Supplement: Supporting Information S1 — Wheat Domestication Accelerated Evolution and Triggered Positive Selection in the β-Xylosidase Enzyme of Mycosphaerella graminicola. Additional information on samples and analyses. (0.04 MB DOC) [file pone.0007884.s001.doc]

**Materials & Methods_S1**

**Source of Isolates;** Sources of wheat-adapted *Mycosphaerella graminicola* isolates included in this study are summarized in Table S1. These *M. graminicola* populations were described in greater detail in previous studies [1], [2], [3], [4], [5].

**Genetic diversity;** Due to the specific questions we addressed in this study, all of the following analyses are based exclusively on exon sequences, although introns were detected in all assessed genes. Summary statistics for population genetic parameters for each gene were calculated using the program DNASP v. 4.10 [6]. To account for the effect of different sample sizes, number of haplotypes and haplotype diversity were adjusted to the smallest sample size using Monte Carlo re-sampling (1000 simulated data sets each) implemented in POPTOOLS v2.6 [7].

**Genetic Coalescent Analysis;** Analyses in BEAST were based on 20 million steps, of which the first two million were discarded as a burn-in period. Parameters were adjusted as suggested by the program until an effective sampling size of at least 300 was achieved for all estimated parameters.

**Deviation from the strict molecular clock;** We used the relaxed clock method [8] implemented in BEAST to test whether the evolutionary rates changed over the genealogy of *M. graminicola*. As outlined in the BEAST manual, evolutionary rates were calculated from the output for each branch using the estimated date of the node of each branch. The calculated evolutionary rates were plotted as a function of time. To compare evolutionary rates with previously published data, the time scale of the resulting coalescent tree was calibrated at the *M. graminicola* and “S1” divergence by assuming a normally distributed root height at this point of 10,000 years bp [9]. For both HYPHY and SLR (PAML) analyses the required phylogenetic tree was constructed with MEGA 4 [10] using the Neighbor-Joining method with the maximum composite likelihood substitution model. Sequences from “S1”, the phylogenetically closest relative to *M. graminicola* identified in a recent study [9], were used for all analyses requiring an outgroup.

**PAML;** A maximum-likelihood method implemented in the program PAML [11], [12] was used to compare different evolutionary models against the data sets. Evidence for positive selection across the entire data set was assessed by comparing the rate of non-synonymous substitutions with the rate of synonymous substitutions (dN /dS = ω) using a phylogenetic analysis based on maximum likelihood as implemented in the PAML software package [13]. Four codon substitution models were compared via likelihood ratio test (LRT). In the first comparison, the nearly neutral model M1 was compared to the alternative model M2. The null model M1 allows for two classes of sites, 0 < ω <1 and ω = 1, while M2 adds ω >1 (indicating positive selection) as a third site class. The second comparison included the likelihood estimates of the neutral null model M7 and the alternative selection model M8. While the site classes for these two models were the same as for the first comparison, they additionally implement a beta distribution for ω.

For “S1” and “S2” the neutral models M1 and M7 fitted the data sets of all enzymes better than the corresponding models M2 and M8, indicating no significant evidence for positive selection using the implemented Bayes Empirical Bayes procedure. In contrast and supporting the results from the McDonald-Kreitman test, strong positive selection (P < 0.001) was inferred for the β-xylosidase data set from wheat-adapted *M*. *graminicola*, whereas likelihood ratio tests for all other genes were non-significant (Table S3). The analyses identified four specific codon sites under strong positive selection with dN/dS > 1 and posterior probabilities > 0.95. The same analysis was conducted separately for the Iranian isolates to compare results between sympatric populations collected from wheat and wild grasses. This analysis identified three of the five codon sites detected in the total *M. graminicola* data set as being under positive selection (Table S3).

**References**

1. McDonald BA, Zhan J, Yarden O, Hogan K, Garton J, et al. (1999) The population genetics of *Mycosphaerella graminicola* and *Stagonospora nodorum*. In: Lucas JA, Bowyer P, Anderson HM, editors. *Septoria* on Cereals: a Study of Pathosystems. New York: CABI Publishing. pp. 44-69.

2. Banke S, Peschon A, McDonald BA (2004) Phylogenetic analysis of globally distributed *Mycosphaerella graminicola* populations based on three DNA sequence loci. Fungal Genet Biol 41: 226-238.

3. Linde CC, Zhan J, McDonald BA (2002) Population structure of *Mycosphaerella graminicola*: From lesions to continents. Phytopathology 92: 946-955.

4. Zhan J, Pettway RE, McDonald BA (2003) The global genetic structure of the wheat pathogen *Mycosphaerella graminicola* is characterized by high nuclear diversity, low mitochondrial diversity, regular recombination, and gene flow. Fungal Genet Biol 38: 286-297.

5. Jürgens T, Linde CC, McDonald BA (2006) Genetic structure of *Mycosphaerella graminicola* populations from Iran, Argentina and Australia. Europ J Plant Pathol 115: 223-233.

6. Rozas J, Sanchez-DelBarrio JC, Messeguer X, Rozas R (2003) DnaSP, DNA polymorphism analyses by the coalescent and other methods. Bioinformatics 19: 2496-2497.

7. Hood GM (2005) PopTools version 2.6.7. Available on the internet. URL http://www.cse.csiro.au/poptools.

8. Drummond AJ, Ho SYW, Phillips MJ, Rambaut A (2006) A relaxed phylogenetics and dating with confidence. PLoS Biol 4: 699-710.

9. Stukenbrock EH, Banke S, Javan-Nikkah M, McDonald BA (2007) Origin and domestication of the fungal wheat pathogen *Mycosphaerella graminicola* via sympatric speciation. Mol Biol Evol 24: 398-411.

10. Tamura K, Dudley J, Nei M, Kumar S (2007) MEGA4: Molecular Evolutionary Genetics Analysis (MEGA) Software Version 4.0. Mol Biol Evol 24: 1596-1599.

11. Nielsen R, Yang Z (1998) Likelihood models for detecting positively selected amino acid sites and applications to the HIV-1 envelope gene. Genetics 148: 929-936.

12. Yang Z, Nielsen R, Goldman N, Pedersen AM (2000) Codon substitution models for heterogeneous selection pressure at amino acid sites. Genetics 155: 431-449.

13. Yang Z (2007) PAML 4: Phylogenetic analysis by maximum likelihood. Mol Biol Evol 24: 1586-1591.
