# Supplementary material for: Wheat Domestication Accelerated Evolution and Triggered Positive Selection in the β-Xylosidase Enzyme of Mycosphaerella graminicola
Source: PLoS One. 2009 Nov 18;4(11):e7884. doi: 10.1371/journal.pone.0007884 (PMC2774967; doi:10.1371/journal.pone.0007884)
Supplement: Table S1 — Origin of fungal isolates. (0.04 MB DOC) [file pone.0007884.s002.doc]

**Table S1.**

| **Pathogen** | **Origin** | **Number of isolates** | **Host plant** | **Year** | **Collector** |
| --- | --- | --- | --- | --- | --- |
| *Mycosphaerella graminicola* | Switzerland | 16 | *Triticum aestivum* | 1999 | McDonald BA |
|  | Germany | 16 | *T. aestivum* | 2004 | von Tiedemann A |
|  | Oregon | 16 | *T. aestivum* | 1990-1992 | Mundt CC & McDonald BA |
|  | Israel | 16 | *T. aestivum* | 1992 | Yarden O |
|  | Iran | 48 | *T. aestivum* | 2001 | Javan-Nikkah M |
|  |  |  |  |  |  |
| *Mycosphaerella spp.*^a^ | Iran | 49 | *Lolium multiflorum* | 2004 | Javan-Nikkah M |
|  | Iran | 3 | *Dactylis glomerata* | 2004 | Javan-Nikkah M |
|  | Iran | 9 | *Agropyron repens* | 2004 | Javan-Nikkah M |

^a^ these samples correspond to the “S1” and “S2” evolutionary sister groups of *M. graminicola*
