# Supplementary material for: Wheat Domestication Accelerated Evolution and Triggered Positive Selection in the β-Xylosidase Enzyme of Mycosphaerella graminicola
Source: PLoS One. 2009 Nov 18;4(11):e7884. doi: 10.1371/journal.pone.0007884 (PMC2774967; doi:10.1371/journal.pone.0007884)
Supplement: Table S2 — Reference sequences of all five plant cell wall degrading enzymes identified from the Mycosphaerella genome project at http://genome.jgi-psf.org. (0.05 MB DOC) [file pone.0007884.s003.doc]

**Table S2.**

| Sequence identification | Enzyme sequence ^a^ | Intron (bp) |
| --- | --- | --- |
| >estExt_fgenesh2_pm.C_10187|Mycgr1  1,4-β-xylosidase | Tctcttcaaaacatcatgtccgatcccaaaccactcatcgagagcgacttctttgcggaccccagcggccgggttttcaacggcagactttatgtttatccgtctcacgatcgccaa  Acagacattcccgacgacgacaatggcagccagtatgatatggctgattaccatgtgctgtcgatgggggagatcggaggtaaagtgacggatcatgggaagatttttgggttggaa  gacgttccgtgggcggcgaagcaactctgg***gctccagactgtgggttcaagaacgacaagtactttttctacttcccagccagggaccacgatggcatctttcgcatcggcgta  gccatcggcgacaaacccgaaggtcctttcaaaccagaacccacggcgattccaggaagctacagcatcgacccggctgtactggtcgacgatgacggcgaagcgtatctctacttc  ggcgggatttggggtggccagctccaatgctggcgcggctcctccttcgacgccgccgcctacgaaacaatggaagccagcggcgatgaacccgccttgatgcctcgcgtcgccaaa  ctcagcccagacatgaagtccttctccggctccgtgcaagatctggtcatcaacgacgccgatggcaagcccctcgccgccgacgaccacgaccgccgcttcttcgaagccgcgtgg  atgcacaaaaaggaagacaagtactacttctcctacagcacgggcgacacgcattacatcgtctacgcgattggcgactcgccggtgggacccttcacgtatgcgggcaggattctc  gagcccgtccagggttggtcgacgcatcattcgattgtggagtttgagggaaagtggtggttgtgttatcatgatgtttcgatttcagggaagaatcatttgagatcggcgaagatc  agggagttgatttatgatgaggaaggcaagatcaagctggcacaggcgcagtcttga | 51 |
| >e_gw.12.751.1|Mycgr1  Cellulase | Gcgggcgtcaccacccggtactgggactgctgcaagccctcctgctcctggcccaacaaagccgccttcagttcccccgtcaaaatctgcggcaaagacgacaacccgatgtccgac  Ccaaacaccgtttccgggtgcgacggcggtccaggtttccagtgttcgaaccagcgctcctgggcgaagactgagagtctggcgtatggattcgcggcggtgaatggagggaatgaa  gccgggtcgtgctgtgcttgttacaaa***ttgaaattcacatccgaagccgccatgggcaaaaccctcgtggtgcaagtcaccaacatcggctacgacgtcggcgccacgcaattc  gacctcgctctgcccggcggcggagtcggaatcttcccccaaggttgccgcaaccaatacggcgtccagaacggctggggttccaacggcgggtacggaggcgtctcctcggaagcg  gagtgcatgacgctgccggcggctctgcaggacggctgcaagttccgcttccagtggttcaagggcagcaacaacccgagtgtgatgtacgagcgggttgcttgcccggcggacttg  gtgagcaggagtggatgtaagagggatgatgattcgcgctttccggcgagcccgtaa | 64 - 90 |
| >estExt_gwp_gw1.C_90520|Mycgr1  Cutinase | ctcctccacaactccaagtccaacccgccgtcatgagaacatctgctttcctcctctcgaccttagccgcatcggcgtttgcgcaggatagcgtacagatcgacctcgctcagatcg  ccaacgcacaaggtagcagtgtacagaagcgcggcattacctccacagagctagagaatggagcttgcaaacaga***tcactttcatctacgctcgcgggtcgacggaacctggta  atatgggcatcgttccaggaccacagacgtgcgacgctttgaagtcccagtacggctcaggcaacgtcgcttgccagggagtggacggaggcaaatacagcgcagatctgttcggca  atttccaaccgaagggaacctatcaggctgccattgacgagggctcgcgactcctcaagcttgccaatagcaagtgtccgaacaccaagatcgttgcaggtggctatagccaaggtg  cggctctcat***ggcaagctccatcagcacactgcccactacagtaatgaaccagatcaagggcgtcgtgctgtatggatacaccaagaacaagcagaatggtggtcgcatcgcga  acttcccgacagagaagacaaaggtcatctgtgaactcggcgacttcgtttgcgatgggacgttgatcatcactattgctcacttgtcgtatctcgatgatgttggcacagcgaaga  gcttttacgttgataggatcaacaatcattag | 105 |
| >e_gw.7.220.1|Mycgr1  Polygalacturonase | atgggtccgaagagctggcccaagcgatctaaagaatgcattgtcccctcaaaagacgatcccacgcacgacgatgctcccgctgtcatcaaggcgttccaggattgttgccatgat  ggccatatcatcttcgagaacaagacataccacatcggctctgtcatgaacaccacgggtctcaaggatgtcgatattgaagtccggggactcctgaaatggtcgacgaacatagac  tactggctcgctcattccatgccgatcggattccagaatcagacatccgcgtgg***catctcggtggtgaggacatccacttctacggccatggccatggtacactcgacggcaac  ggccaagtttggtacgattttgccaaaggagtcagcaacatccacgggaggccccaccagatcaccatcacgaacaccaagaacagcgtcatcgagggtttaagattcgtacagtct  cagatgtggacaatgacggtcgcgcgctcagagaaagtcctactccaagacatctacgtctcaagtaccagcacggatccagctattcgcagtaatgtcaataccgacggttgcgac  acagtttacaccaacgacatcaccttcctgcgctggacaatcaccaacggcgacgacagcatctccatgaagcagaatagtaccaacatctacatctccaactgtaccttctacaac  ggtgcttctcttgcgatgggcagcatcggacaataccctggacaaattgagatcatcgagaacatcaccgccacggacatcaagatgatcaataccggctacgcaggccgcatcaag  acttgggtcgggaagaacaagggtttccctccgaacggcggcggaggtggactaggccatgcgaagaacattacctttcgtaattttgagctcgagggcgttggaaccgcctggctc  atcacgcagtgtacattctacgatggaccggaaaacgccagagaggtacaccaagaagccgaaccggactgtacgaactcccaatttgagatctccgacctcaactggggcgacacg  cacggcacgattcgctcagaacgcatcgcagcgctgcaatgctccgctacgaagccctgccacaacatcaatatcttcaacaacagcttgacggctttggatacgaagaagccggcc  gagacatttctctgcgaacaagtgaaggatacgtgtgggttcacgtgcacggaggagtgcaatggaaggtgtccccgttcttga | 91 |
| >estExt_gwp_gw1.C_40514|Mycgr1  Xylanase | cttcatctttcagcttcgagatcaaacaaagtcatgctcttcacacgcgctctgctcggcctcgccgccgctacgtactctctggccgctcccatcaccgaggcctctcccaacccg  gactcggccacccacctcttcgctcgcgagagcccgggcactggcacgaacaatggctactactactccttctggaccgacggtggaggccaagtgaactacaacaatggcccgggc  ggctcgtacacggtctcgtggagcaacgttgggaacttcgtt***gcgggcaagggttgggctacagggtctgctagaaccatcaactactccggctccttcaacccctccggcaac  ggctacatcgccgcctacggctggacccgcaaccctctgatcgaatactacgtcgtcgaatcctacggcacctacaaccccagctct***gcggctcagaagaagggaaccgtcacc  accgacggcggcacctacgatatcctccaaaccacccgcgtcaaccagccctccattgacggcacgcagacctttcagcaattctggagcgtgcgccagcagaagcgcgtcggcggc  acggtgaccatgaagaaccacttcgacgcttggtcgaggctgggcatgaagctgggttcgtcgcagaactacc***agattttggcgaccgagggataccagagcagcgggtctgcg  agcatcaccgtttcgtagattggatgtggttgaggaagtgtttttcgcgtgttgc | 134 - 286 |

^a^ Primer sites are underlined; asterisks denote intron region
