# Supplementary material for: Wheat Domestication Accelerated Evolution and Triggered Positive Selection in the β-Xylosidase Enzyme of Mycosphaerella graminicola
Source: PLoS One. 2009 Nov 18;4(11):e7884. doi: 10.1371/journal.pone.0007884 (PMC2774967; doi:10.1371/journal.pone.0007884)
Supplement: Table S3 — Summary statistics for likelihood-ratio-test (LRT) using PAML for positive selection in the Mycosphaerella graminicola samples from wheat. (0.05 MB DOC) [file pone.0007884.s004.doc]

**Table S3.**

|  |  |  |  |  |  |  |  |  |  |  |
| --- | --- | --- | --- | --- | --- | --- | --- | --- | --- | --- |
|  |  | -lnL |  |  |  |  | P value |  | Selected codons ^a^ |  |
| Enzyme | M1 | M2 | M7 | M8 |  | M2 vs. M1 | M8 vs. M7 |  | Site | Probability |
|  |  |  |  |  |  |  |  |  |  |  |
| β-Xylosidase | 1714 | 1699 | 1718 | 1702 |  | < 0.001 | < 0.001 |  | 84 | 0.997 |
|  |  |  |  |  |  |  |  |  | 192 * | 0.999 |
|  |  |  |  |  |  |  |  |  | 222 * | 1.000 |
|  |  |  |  |  |  |  |  |  | 246 * | 0.999 |
|  |  |  |  |  |  |  |  |  |  |  |
| Cellulase | 1237 | 1236 | 1237 | 1238 |  | > 0.1 | > 0.1 |  | - | - |
| Cutinase | 1541 | 1541 | 1542 | 1543 |  | > 0.1 | > 0.1 |  | - | *-* |
| Polygalacturonase | 2141 | 2142 | 2141 | 2141 |  | > 0.1 | > 0.1 |  | - | - |
| Xylanase | 2451 | 2450 | 2450 | 2449 |  | > 0.1 | > 0.1 |  | - | - |
|  |  |  |  |  |  |  |  |  | - | - |

NOTE. No significant P values for the model comparisons and no selected codon sites were detected for the wild grass samples “S1” and “S2”.

^a^ only selected codons with P > 0.95 are listed

*) Asterisks denote significant positive sites for the Iran samples on wheat.
