# Supplementary material for: Wheat Domestication Accelerated Evolution and Triggered Positive Selection in the β-Xylosidase Enzyme of Mycosphaerella graminicola
Source: PLoS One. 2009 Nov 18;4(11):e7884. doi: 10.1371/journal.pone.0007884 (PMC2774967; doi:10.1371/journal.pone.0007884)
Supplement: Table S4 — McDonald-Kreitman test of neutrality for Mycosphaerella graminicola nucleotide data sets. (0.05 MB DOC) [file pone.0007884.s005.doc]

**Table S4.**

|  |  |  |  |  |  |  |  |  |
| --- | --- | --- | --- | --- | --- | --- | --- | --- |
|  |  | Polymorphic |  |  | Fixed |  |  |  |
| Enzyme | *n* | Synonymous | Non-synonymous |  | Synonymous | Non-synonymous | NI^a^ | Probability |
|  |  |  |  |  |  |  |  |  |
| β-Xylosidase | 95 | 24 | 19 |  | 41 | 11 | 2.951 | 0.004 |
| Cellulase | 108 | 46 | 9 |  | 27 | 21 | 2.225 | 0.025 |
| Cutinase | 109 | 36 | 9 |  | 18 | 2 | 0.221 | 0.479 |
| Polygalacturonase | 108 | 35 | 18 |  | 7 | 5 | 0.720 | 0.741 |
| Xylanase | 101 | 37 | 13 |  | 21 | 14 | 0.527 | 0.237 |
|  |  |  |  |  |  |  |  |  |

NOTE. *Mycosphaerella* spp. collected from wild grasses were taken as outgroup. Probability values for deviation from the neutrality model were obtained using a two-tailed Fisher’s exact test

^a^ Neutrality-Index
