# Supplementary material for: Wheat Domestication Accelerated Evolution and Triggered Positive Selection in the β-Xylosidase Enzyme of Mycosphaerella graminicola
Source: PLoS One. 2009 Nov 18;4(11):e7884. doi: 10.1371/journal.pone.0007884 (PMC2774967; doi:10.1371/journal.pone.0007884)
Supplement: Table S5 — Likelihood ratio testing for the assumption of a molecular clock. (0.03 MB DOC) [file pone.0007884.s006.doc]

**Table S5.**

|  |  | **dN** |  |  |  | **dS** |  |  |  | **full** |  |
| --- | --- | --- | --- | --- | --- | --- | --- | --- | --- | --- | --- |
| **PCWDE** | **2ΔLR** | **df** | ***P*** |  | **2ΔLR** | **df** | ***P*** |  | **2ΔLR** | **df** | ***P*** |
| β-Xylosidase | 72.5 | 32 | <0.001 |  | 82.3 | 32 | <0.001 |  | 161.1 | 64 | <0.001 |
| Cellulase | 6.4 | 30 | >0.1 |  | 7.4 | 30 | >0.1 |  | 10.2 | 60 | >0.1 |
| Cutinase | 4.1 | 28 | >0.1 |  | 3.1 | 28 | >0.1 |  | 15.1 | 56 | >0.1 |
| Polygalacturonase | 2.8 | 32 | >0.1 |  | 4.5 | 32 | >0.1 |  | 12.4 | 64 | >0.1 |
| Xylanase | 8.2 | 30 | >0.1 |  | 7.1 | 30 | >0.1 |  | 13.6 | 30 | >0.1 |
