# Supplementary figures and images for: Wheat Domestication Accelerated Evolution and Triggered Positive Selection in the β-Xylosidase Enzyme of Mycosphaerella graminicola
Source: PLoS One. 2009 Nov 18;4(11):e7884. doi: 10.1371/journal.pone.0007884 (PMC2774967; doi:10.1371/journal.pone.0007884)

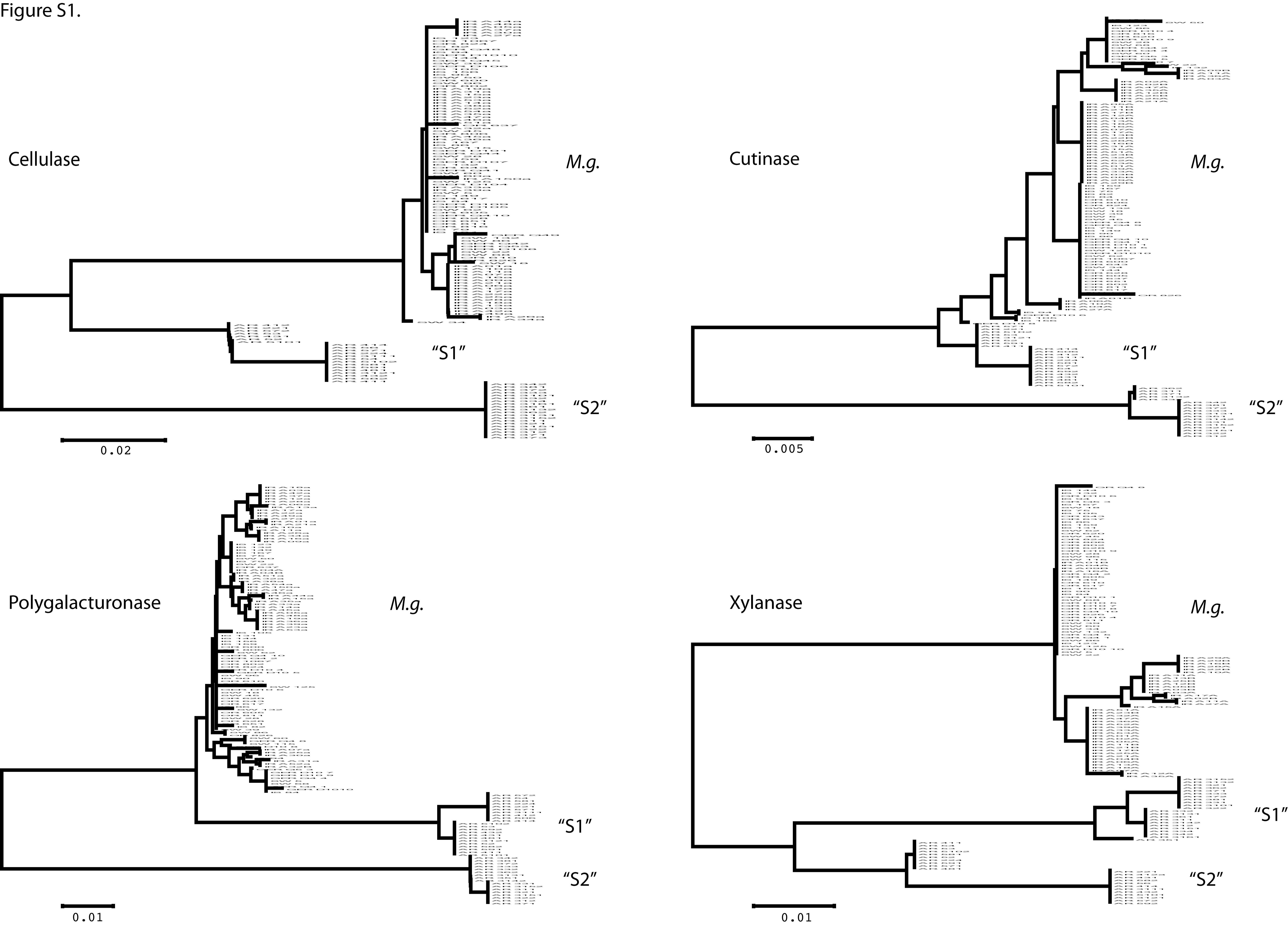

Supplement: Figure S1 — Phylogenetic relationships among fungal samples for different enzymes. Neighbor-Joining trees (NJ) based on the PAM matrix (Dayhoff model) of amino acid sequences for the cell-wall-degrading enzymes cellulase, cutinase, polygalacturonase and xylanase. (1.07 MB TIF) [file pone.0007884.s007.tif]
